# Accelerated structural valve deterioration in systemic sclerosis patients following transcatheter aortic valve replacement: a case series

**DOI:** 10.1093/ehjcr/ytaf060

**Published:** 2025-02-05

**Authors:** Yuval Avidan, Joy Feld, Amir Aker, Ronen Jaffe

**Affiliations:** Department of Cardiology, Lady Davis Carmel Medical Center, 7 Michal St., Haifa 3436212, Israel; Department of Rheumatology, Lady Davis Carmel Medical Center, Haifa 3436212, Israel; Department of Cardiology, Lady Davis Carmel Medical Center, 7 Michal St., Haifa 3436212, Israel; Department of Cardiology, Lady Davis Carmel Medical Center, 7 Michal St., Haifa 3436212, Israel

**Keywords:** Systemic sclerosis, Aortic stenosis, Transcatheter aortic valve replacement, Structural valve deterioration, Case series

## Abstract

**Background:**

Systemic sclerosis (SSc) is marked by an excessive systemic accumulation of collagen. Recent literature implies that aortic stenosis is more prevalent in patients with SSc than previously thought. While there are limited feasibility studies on transcatheter aortic valve replacement (TAVR) in this population, the long-term outcomes remain uncertain.

**Case summary:**

We report two cases of patients with SSc who developed early structural valve deterioration following TAVR, necessitating successful redo-TAVR procedures. Both patients exhibited extensive soft tissue calcinosis as a manifestation of their underlying condition.

**Discussion:**

The fibrotic and calcific processes inherent to certain SSc subtypes could potentially adversely impact the durability and functionality of transcatheter aortic valves. Our observation highlights the need for vigilant post-procedural surveillance and individualized management strategies in this unique patient population. Further investigation into the mechanisms underlying valve degeneration in this patient subset is warranted. Nevertheless, redo-TAVR procedure appears to be a viable option.

**Learning points**

- Patients with systemic sclerosis may experience expedited degeneration of transcatheter aortic valves.
- The underlying disease processes of systemic sclerosis may contribute to structural valve deterioration.
- Increased frequency of echocardiographic monitoring is justifiable for patients with systemic sclerosis following TAVR.

## Introduction

Systemic sclerosis (SSc) is a connective tissue disorder characterized by the excessive build-up of collagen in the skin and internal organs.^1^ Valvular involvement is not uncommon,^2^ with recent studies indicating that aortic stenosis (AS) is more prevalent than previously thought.^3–5^ Before the era of transcatheter aortic valve replacement (TAVR), these patients were commonly considered unsuitable for surgical aortic valve replacement (SAVR) due to their co-morbidities.^6^ Consequently, information on SAVR-related outcomes in this population is limited. Implantation of a transcatheter aortic valve (THV) presents a potential therapeutic option for patients who are at high surgical risk.^7^ Feasibility studies involving small SSc cohorts undergoing TAVR have been published, though long-term outcome data are lacking.^8–10^

Bioprosthetic valve dysfunction has been classified as structural valve deterioration (SVD), non-structural valve dysfunction, clinical valve thrombosis, or endocarditis according to Valve Academic Research Consortium-3 criteria.^11,12^ In a low-risk cohort, the 10-year risk of severe SVD was 1.5% and 10.0% after TAVR and SAVR, respectively, and the risk of severe NSVD was 20.5% and 43.0%.^13^ In cases of a failing prosthetic valve, redo-TAVR, with implantation of a second transcatheter heart valve (THV-in-THV), can be performed.

We present two cases of SSc patients who experienced early SVD following TAVR, necessitating subsequent THV-in-THV procedure. We discuss potential underlying pathophysiological mechanisms that could possibly explain this clinical scenario.

## Summary figure

**Figure:**
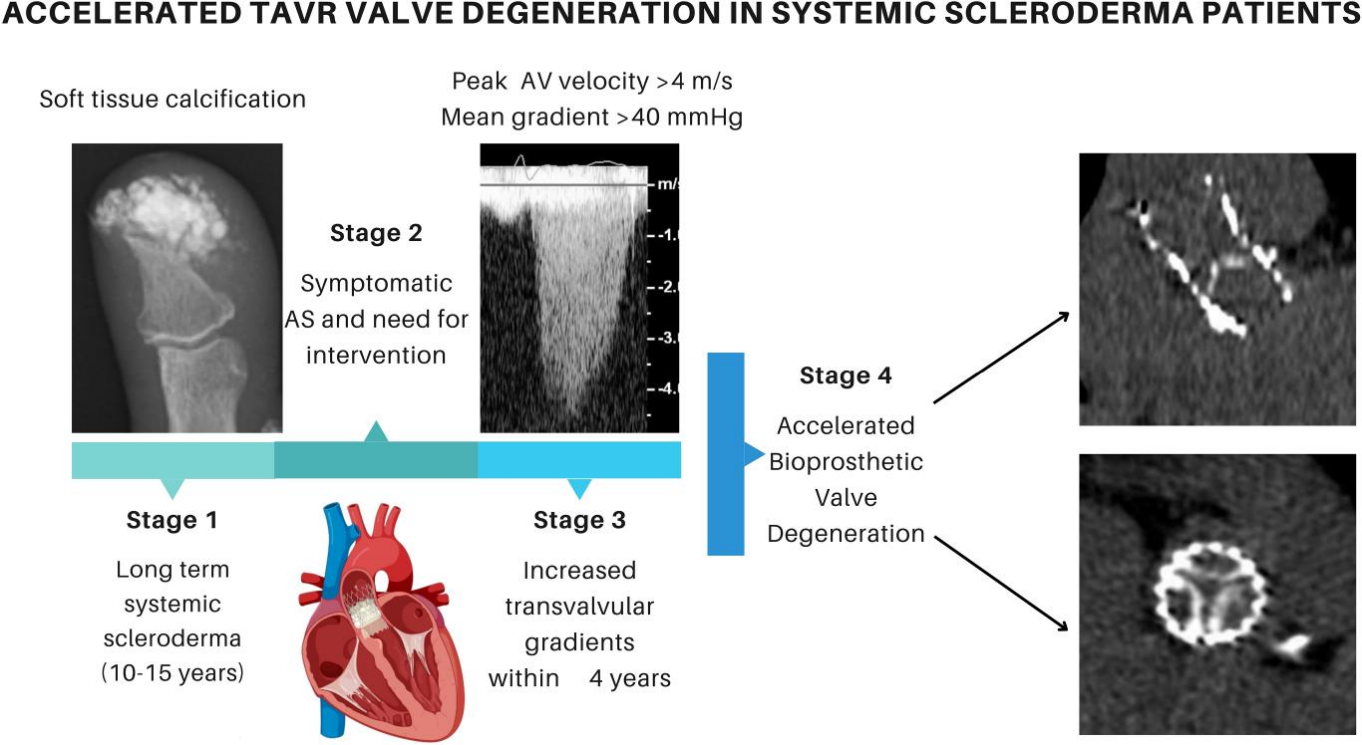


## Patient 1

**Figure 1:**
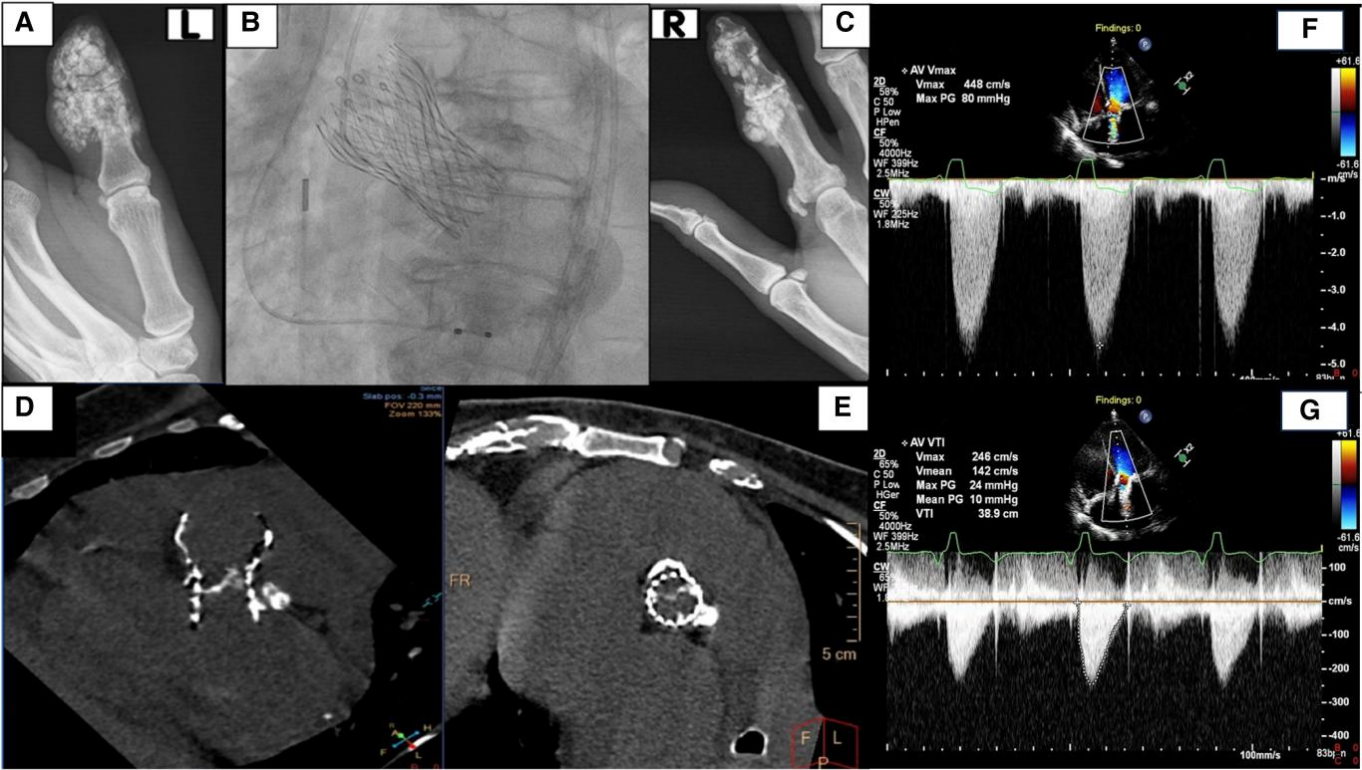
(*A*, *C*) Plain radiographs showing subcutaneous and periarticular calcification of the left and right hands, respectively. (*B*) Right anterior oblique view demonstrating the final outcome of a TAV-in-TAV procedure with two overlapping self-expandable valves. (*D*, *E*) CT coronal and axial views of a degenerated self-expandable valve illustrating thickening and calcification of the valve leaflets. (*F*, *G*) Continuous wave Doppler recording from transthoracic apical five-chamber view showing transvalvular peak jet velocity before and after redo-TAVR.

A 64-year-old woman presented with symptoms of heart failure secondary to severe SVD of a 26-mm self-expanded CoreValve (Medtronic), which had been implanted 4 years previously due to severe symptomatic AS. Transthoracic echocardiography (TTE) following the index procedure documented normal haemodynamics of the THV. Her medical history included long-standing limited SSc, treated with daily hydroxychloroquine (200 mg daily), methotrexate (15 mg weekly), and intravenous iloprost on demand. The patient’s immunosuppressant therapy remained unchanged in recent years. The condition was marked by Raynaud’s phenomenon, digital ulcers, oesophageal dysmotility, and pulmonary arterial hypertension. Another prominent manifestation of her disease was extensive soft tissue calcinosis in her hand (*Figure 1A* and *C*). Serological testing was positive for antinuclear antibodies, but negative for anti-centromere antibodies (ACA) and scleroderma 70 antibodies. Additionally, she had coronary artery disease and had undergone left main bifurcation stenting.

Physical examination revealed a grade 4/6 ejection systolic murmur with a soft second heart sound and no evidence of fluid overload. Transthoracic echocardiography confirmed prosthetic valve stenosis, with peak/mean THV gradients of 80/49 mmHg, peak aortic flow velocity of 4.48 m/s (*Figure 1F*; Supplementary material online, *Video S1*), and dimensionless index (DI) of 0.23 (reference values suggestive of severe stenotic prosthetic aortic valve; peak velocity > 4 m/s, mean gradient ≥ 40 mmHg or a ≥20 mmHg change from baseline, or DI < 0.25^12,14^). Due to the inconclusive TTE findings regarding the mechanism of valve deterioration and the impracticality of transoesophageal echocardiography due to microstomia and oesophageal pathology, multidetector computerized tomography (MDCT) was performed, revealing basal calcification of the bioprosthetic aortic valve leaflets, which impeded their movement (*Figure 1D* and *E*). She underwent a THV-in-THV procedure using intracardiac echocardiography guidance, with implantation of a 26 mm Evolut R valve via transfemoral approach (*Figure 1B*). Post-TAVR TTE demonstrated optimal haemodynamics of the THV with minimal paravalvular leak (PVL, *Figure 1G*; Supplementary material online, *Video S2*). During the postoperative period, she received blood transfusions and underwent a gastrointestinal endoscopy, which did not identify a source of bleeding. She was discharged 5 days later. Five months later, the patient developed acute coronary syndrome and subsequently passed away.

## Patient 2

**Figure 2:**
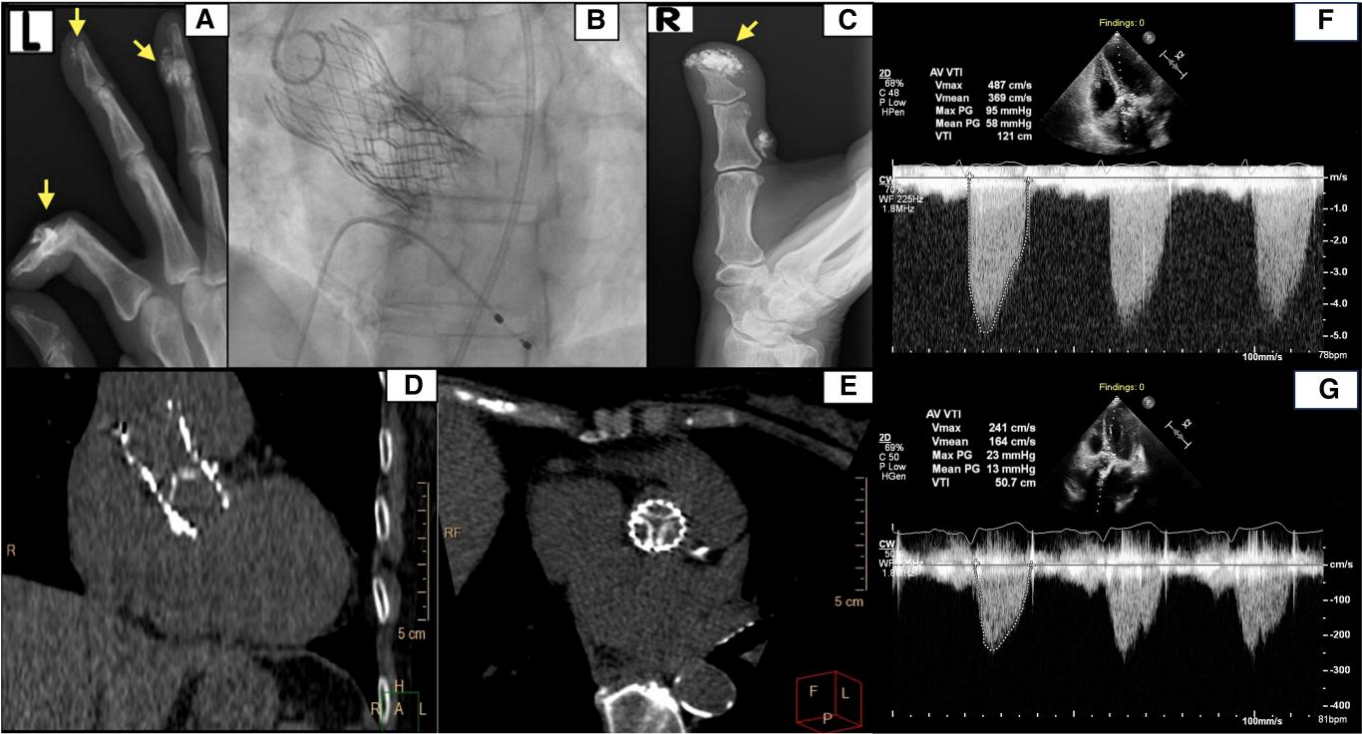
(*A*, *C*) Plain radiographs showing subcutaneous and periarticular calcification of the left and right hands, marked by arrows. (*B*) Right anterior oblique view illustrating the final outcome of a TAV-in-TAV procedure with a balloon-expandable valve positioned within a previously implanted self-expandable valve. (*D*, *E*) CT coronal and axial views of a degenerated self-expandable valve demonstrating thickening and calcification of valve leaflets. (*F*, *G*) Continuous wave Doppler recording from transthoracic apical five-chamber view showing transvalvular peak jet velocity before and after redo-TAVR.

A 72-year-old woman underwent TAVR due to severe symptomatic AS, with implantation of a 26 mm Evolut-Pro valve (Medtronic). Post-procedural TTE documented normal THV haemodynamics with a peak pressure gradient of 16 mmHg across the prosthetic valve and no PVL. She presented 4 years later with new-onset exertional dyspnoea, due to severe SVD with THV stenosis. Her medical history included long-standing limited SSc, treated with daily hydroxychloroquine (200 mg daily) and azathioprine (50 mg twice daily) for the past several years, manifested by substantial digital ulceration and calcinosis in her hands (*Figure 2A* and *C*). She was seropositive for ACA. Her examination was notable for a loud ejection systolic murmur with an absent second heart sound and mild fluid retention. Transthoracic echocardiography measured a peak/mean THV pressure gradient of 95/58 mmHg, peak aortic flow velocity of 4.87 m/s, and a DI of 0.21 (*Figure 2F*; Supplementary material online, *Video S3*). The MDCT revealed severely calcified bioprosthetic valve with reduced mobility (*Figure 2D* and *E*). A 23 mm Sapien S3 valve was implanted within the existing bioprosthesis through transfemoral vascular access (*Figure 2B*). Subsequent TTE indicated normal THV function and trivial PVL (*Figure 2G*; Supplementary material online, *Video S4*). She was discharged 3 days later. Echocardiographic evaluation conducted 6 months after the redo-TAVR revealed normal haemodynamics. The patient was doing well at the 9-month follow-up.

## Discussion

Understanding of the pathophysiological mechanisms leading to AS and SVD within a prosthetic valve is incomplete and beyond the scope of this paper. Current data suggest that patients with a long-standing limited subtype of SSc and seropositivity for ACA are more prone to develop AS^8^; however, the underlying mechanism remains unclear. Intriguing insights emerge from histopathological examination of specimens obtained from three SSc patients who underwent SAVR: one patient had visible calcium deposition, another had substantial fibrin and platelet deposits, and the third exhibited primarily acellular fibrosis with minimal calcification, unlike cases of traditional degenerative AS.^6,15^ Although bicuspid aortic valve (BAV) morphology is common in patients under 70 who present with AS,^16^ cohorts of SSc patients with severe AS reported mean ages of 67 (*n* = 6), 70 (*n* = 5), and 70 (*n* = 13), all of whom presented with tri-leaflet aortic valves.^8–10^ Similarly, an early occurrence in the SSc population was described by Ferrari *et al.*^6^ and Sponga *et al.*,^15^ as well as our subjects, who were 60 and 67 during the initial TAVR, none of whom had a BAV to account for the early AS.

These findings collectively suggest a possible distinct pathogenesis in which underlying connective tissue disease may contribute to, or accelerate the progression of AS. Several mechanisms may be involved; chronic inflammation is proposed to play a pivotal role in the development and advancement of valvular heart disease, particularly AS, as observed in patients with rheumatoid arthritis and psoriasis. These have an elevated risk of AS development that cannot be solely explained by traditional cardiovascular risk factors.^3^ Likewise, in SSc, the widespread immune system activation, endothelial dysfunction, and microvascular vasculopathy may potentially contribute to this process.^9^ Women with AS tend to exhibit more pronounced valvular fibrosis rather than calcification.^3^ Considering the female predominance in SSc, it is possible that this entity contributes to accelerated fibrotic changes in this context. Lastly, both of our patients suffered from extensive cutaneous calcinosis. Considering this, it is important to note that ectopic calcification, often observed in advanced chronic renal failure, has been suggested to implicate AS progression.^17^ A study found that moderate or severe SVD is significantly more common in dialysis patients who underwent aortic valve replacement than in non-dialysis patients (29% vs. 5% at 5 years, *P* < 0.001).^17^ We therefore postulate that such extensive calcinosis in scleroderma could potentially influence the progression of valvular calcification.

Transcatheter heart valve durability is central to patient management following TAVR. Nevertheless, paucity of randomized data,^12,18^ variations in valve types, and heterogeneous definitions of SVD,^12^ along the fact that many TAVR recipients are octogenarians,^12^ hinder the attempts to establish a universal definition for long-term durability. Furthermore, as the optimal treatment options for redo-TAVR are continuously evolving, given their prevailing co-morbidities, THV-in-THV in SSc patients presents an even greater challenge. As evident from both cases, the perioperative stay was short and free from major complications.

## Conclusion

We described two SSc patients who experienced rapid SVD following TAVR and successfully underwent THV-in-THV intervention. One could hypothesize that underlying SSc played a pivotal role in this process. Further research addressing the specific mechanisms that contribute to valve degeneration in these patients is necessary before firm conclusions can be drawn. Nevertheless, if this presumed explanation does indeed contribute to valve degeneration in this specific subgroup, it raises questions about the appropriateness of TAVR in these patients.

## Supplementary Material

ytaf060_Supplementary_Data

## Data Availability

The data would be available anonymized upon reasonable request to the corresponding author.

## Lead author biography

Dr Avidan is currently a Cardiology Fellow at Carmel Hospital, Haifa, Israel. He completed internal medicine residency in the same hospital.

## Supplementary material

**Consent:** The patients or next-of-kin gave informed consent, in line with COPE guidelines for the present manuscript.

**Funding:** None declared.

## References

[1] Volkmann ER , Andréasson K, Smith V. Systemic sclerosis. Lancet 2023;401:304–318. 36442487 10.1016/S0140-6736(22)01692-0 PMC9892343

[2] Colaci M , Schinocca C, Bosco YD, Ronsivalle G, Guggino G, de Andres I, et al Heart valve abnormalities in systemic sclerosis patients. JCR: J Clin Rheumatol 2022;28:e95–e101. 33252390 10.1097/RHU.0000000000001638

[3] Kurmann RD , El-Am EA, Radwan YA, Sandhu AS, Crowson CS, Matteson EL, et al Increased risk of valvular heart disease in systemic sclerosis: an underrecognized cardiac complication. J Rheumatol 2021;48:1047–1052. 33452164 10.3899/jrheum.201005 PMC8254733

[4] Butt SA , Jeppesen JL, Torp-Pedersen C, Sam F, Gislason GH, Jacobsen S, et al Cardiovascular manifestations of systemic sclerosis: a Danish nationwide cohort study. J Am Heart Assoc 2019;8:e013405. 31446827 10.1161/JAHA.119.013405 PMC6755829

[5] Movahed MR , Timmerman B, Hashemzadeh M. Independent association of aortic stenosis with many known cardiovascular risk factors and many inflammatory diseases. Arch Cardiovasc Dis 2023;116:467–473. 37749002 10.1016/j.acvd.2023.07.008

[6] Ferrari G , Pratali S, Pucci A, Bortolotti U. Aortic valve replacement in systemic sclerosis. J Cardiovasc Med 2015;16:S60–S61. 10.2459/JCM.0b013e328365aa9d 24625565

[7] Leon MB , Smith CR, Mack M, Miller DC, Moses JW, Svensson LG, et al Transcatheter aortic-valve implantation for aortic stenosis in patients who cannot undergo surgery. N Engl J Med 2010;363:1597–1607. 20961243 10.1056/NEJMoa1008232

[8] Balbir-Gurman A , Braun-Moscovici Y. AB0549 the relevance of aortic stenosis and outcome of TAVI procedure for valve repair in scleroderma patients. Ann Rheum Dis 2020;79:1571.3–151571.

[9] Bernelli C , Chieffo A, Giustino G, Montorfano M, Latib A, Panoulas VF, et al Preliminary outcomes after transcatheter aortic valve implantation in patients with systemic sclerosis. EuroIntervention 2015;10:1464–1467. 25912392 10.4244/EIJV10I12A255

[10] Alman K , Sadd CJ, Ravel A, Raza F, Chybowski A, Runo JR. Prevalence of aortic stenosis and TAVR outcomes in patients with systemic sclerosis-associated pulmonary hypertension. Pulm Circ 2022;12:e12118. 36034401 10.1002/pul2.12118 PMC9400580

[11] Généreux P , Piazza N, Alu MC, Nazif T, Hahn RT, Pibarot P, et al Valve Academic Research Consortium 3: updated endpoint definitions for aortic valve clinical research. Eur Heart J 2021;42:1825–1857. 33871579 10.1093/eurheartj/ehaa799

[12] Capodanno D , Søndergaard L. How to define durability of transcatheter and surgical bioprosthetic aortic valves. JACC Cardiovasc Interv 2020;13:257–260. 31973798 10.1016/j.jcin.2019.11.025

[13] Thyregod HGH , Jørgensen TH, Ihlemann N, Steinbrüchel DA, Nissen H, Kjeldsen BJ, et al Transcatheter or surgical aortic valve implantation: 10-year outcomes of the NOTION trial. Eur Heart J 2024;45:1116–1124. 38321820 10.1093/eurheartj/ehae043 PMC10984572

[14] Zoghbi WA , Jone PN, Chamsi-Pasha MA, Collins CT, Desai KA, Y M, et al Guidelines for the evaluation of prosthetic valve function with cardiovascular imaging: a report from the American Society of Echocardiography developed in collaboration with the Society for Cardiovascular Magnetic Resonance and the Society of Cardiovascular Computed Tomography. J Am Soc Echocardiogr 2024;37:2–63. 38182282 10.1016/j.echo.2023.10.004

[15] Sponga S , Basso C, Ruffatti A, Gerosa G. Systemic sclerosis and aortic valve stenosis: therapeutic implications in two cases of aortic valve replacement. J Cardiovasc Med 2009;10:560–562. 10.2459/JCM.0b013e32832c1726 19384239

[16] Carabello BA , Paulus WJ. Aortic stenosis. Lancet 2009;373:956–966. 19232707 10.1016/S0140-6736(09)60211-7

[17] Kuroda Y , Marui A, Arai Y, Nagasawa A, Tsumaru S, Arakaki R, et al Impact of dialysis in patients undergoing bioprosthetic aortic valve replacement. Interact Cardiovasc Thorac Surg 2021;33:348–353. 33961031 10.1093/icvts/ivab106 PMC8691590

[18] Ler A , Ying YJ, Sazzad F, Choong AMTL, Kofidis T. Structural durability of early-generation Transcatheter aortic valve replacement valves compared with surgical aortic valve replacement valves in heart valve surgery: a systematic review and meta-analysis. J Cardiothorac Surg 2020;15:127. 32513222 10.1186/s13019-020-01170-7 PMC7278207

